# Influence of CoFeB layer thickness on elastic parameters in CoFeB/MgO heterostructures

**DOI:** 10.1038/s41598-023-37808-4

**Published:** 2023-07-01

**Authors:** S. Shekhar, S. Mielcarek, Y. Otani, B. Rana, A. Trzaskowska

**Affiliations:** 1 Institute of Spintronics and Quantum Information, Faculty of Physics, Adam Mickiewicz University, Uniwersytetu Poznańskiego 2, 61-614 Poznan, Poland; 2 Center for Emergent Matter Science, RIKEN, 2-1 Hirosawa, Wako, 351-0198 Japan; 3 Institute for Solid State Physics, University of Tokyo, Kashiwa, Chiba 277-8581 Japan

**Keywords:** Materials science, Magnetic properties and materials, Surfaces, interfaces and thin films

## Abstract

The surface acoustic waves, i.e., surface phonons may have huge potential for future spintronic devices, if coupled to other waves (e.g., spin waves) or quasiparticles. In order to understand the coupling of acoustic phonons with the spin degree of freedom, especially in magnetic thin film-based heterostructures, one needs to investigate the properties of phonons in those heterostructures. This also allows us to determine the elastic properties of individual magnetic layers and the effective elastic parameters of the whole stacks. Here, we study frequency versus wavevector dispersion of thermally excited SAWs in CoFeB/MgO heterostructures with varying CoFeB thickness by employing Brillouin light spectroscopy. The experimental results are corroborated by finite element method-based simulations. From the best agreement of simulation results with the experiments, we find out the elastic tensor parameters for CoFeB layer. Additionally, we estimate the effective elastic parameters (elastic tensors, Young’s modulus, Poisson’s ratio) of the whole stacks for varying CoFeB thickness. Interestingly, the simulation results, either considering elastic parameters of individual layers or considering effective elastic parameters of whole stacks, show good agreement with the experimental results. These extracted elastic parameters will be very useful to understand the interaction of phonons with other quasiparticles.

## Introduction

Surface acoustic waves (SAWs) are elastic waves that propagate along the surface of elastic materials with their displacement amplitude decaying with depth into the materials, so that the energy of acoustic phonons, associated with SAWs, is mostly confined in the vicinity of the surface. There are many types of SAWs such as Rayleigh waves, Sezawa waves, Pseudo-SAWs, Lamb waves, Love waves, and so on. One of the most used SAWs in modern devices is the Rayleigh waves, which is named after Lord Rayleigh who first reported the propagation and properties of SAWs in 1885^1^. Today, SAWs are commonly used in microelectronics devices, sensors and filters because of their low power consumption, high sensitivity and broad tunability of operational frequency. SAW-based sensors can be utilized for characterizing various physical (e.g., density, viscosity) and chemical properties of materials^2^. Moreover, these sensors can be used to sense micro-pressures^3^ and to detect bacteria spores such as Bacillus thuringiensis and E. coli^4^. Apart from these, SAWs also promise to develop future spintronics devices if coupled to other waves, e.g., spin waves (SWs), i.e., the collective precessional motion of ordered magnetic spins in magnetic materials. It has been demonstrated that the SAWs can be a very useful tool for exciting^5–9^ and manipulating^10–12^ SWs and vice versa^13–15^, which can be a compliment to current CMOS based technology. Furthermore, SAWs have also proven to be a potential tool for the nucleation of magnetic skyrmions^16^, the creation of magnonic crystals, i.e., artificial crystals for tailoring magnonic band structures^17,18^, domain wall driving^19,20^, spin current generator^21^, nano-oscillator based reservoir computation^22^. Recently, magnetic thin films and their heterostructures are promising to be potential candidates for future spintronics applications because of their fascinating interfacial properties. Therefore, it is quite essential to understand how acoustic phonons couple with SWs, i.e., magnons and spin degree of freedom, especially, in magnetic thin film heterostructures. At the same time, it is also important to increase the coupling efficiency between phonons and spin degree of freedom.

A significant amount of research has been dedicated to the investigation of the coupling between magnons and acoustic phonons in magnetic thin films. It was observed that the magnetic anisotropies and spatial profiles of SWs and acoustic phonons play a crucial role in the coupling phenomena^23–26^. Generally, the coupling strength can be increased by maximizing the overlapping area of magnon dispersion curves with the phonon dispersion curves. This can be achieved by tuning the dispersion curves for phonons and magnons by playing with the elastic and magnetic parameters of the materials, as properties of acoustic phonons and magnons strongly depend upon the elastic and magnetic parameters of the materials, respectively. CoFeB thin films are known to be one of the most promising ferromagnetic materials for future spintronics devices due to compelling features such as low Gilbert damping^27^, negligible magneto crystalline anisotropy, high tunnel magnetoresistance^28^ and large spin polarization^29^. They have shown potential for many applications like magnetic tunnel junctions^27^, racetrack memory^30^, magnetic random-access memory^31^, read head^32^, and so on. Therefore, it will be quite promising to investigate magnon–phonon coupling in CoFeB thin films and their heterostructures. While doing so, one should separately investigate phonon and magnon dispersion relations in CoFeB thin film-based heterostructures as a first step. Hence, we have adapted CoFeB/MgO heterostructures in this study for the investigation of phonon dispersion. This is stimulated by the fact that the elastic parameter of one thin layer may be drastically different from its bulk values. Moreover, the effective elastic property of the whole stacks could also be very different than the single layer.

The most used technique to study SAWs and estimate the elastic properties of thin films is Brillouin light spectroscopy (BLS). This method allows us to measure acoustic phonons in a non-invasive manner and provides information about the frequency and wavevector of SAWs. Several studies have been performed using the BLS technique on the estimation of elastic parameters for different thin films such as ZnO^33^, SnO_2_ and SnS_2_^34^, ITO^35^, TiN^36^; as well as for magnetic multilayers such as [Ni_80_Fe_20_/Au/Co/Au]_10_^37^, [CoFeB/Au]_N_^38^ and topological insulators^39^. Trzaskowska et al*.* observed that the change in the effective thickness of the layer (by varying the number of the repetitive layer) has an influence on the dispersion relation of the SAWs leaving the profiles of the elastic wave to be the same^38^. It was also observed that for multilayered thin films, the elastic properties strongly depend on their synthesis condition^40–42^, materials of the deposited layers, and the type of substrate^43,44^. Hence, estimating these elastic properties experimentally as well as theoretically is very important from the application point of view. In this study, we investigate the dispersion relation (the frequency versus wavevector) of SAWs in CoFeB/MgO heterostructures with varying CoFeB thickness by employing BLS. Finite element method (FEM) based simulations are performed to corroborate experimental results. From the best agreement of simulation results with the experiments we find out the elastic tensor parameters for CoFeB layer and estimate the effective elastic parameters of the whole stacks for varying CoFeB thickness.

## Material and methods

### Sample fabrication

**Figure 1:**
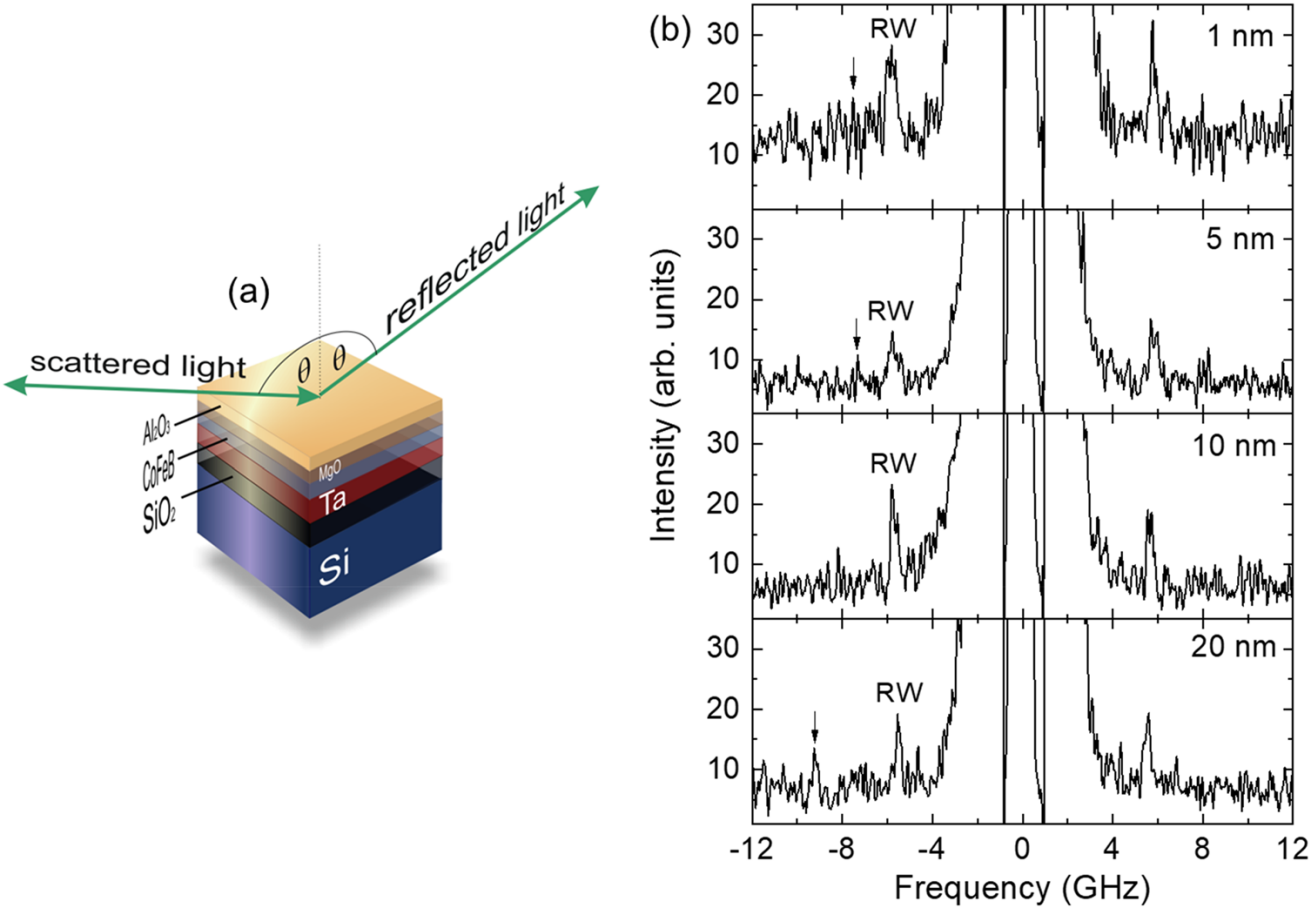
(**a**) The schematic view of the studied samples. The green lines represent the geometry of the experiment. (**b**) The BLS spectra obtained from the Si/SiO_2_/Ta/CoFeB(*t*)/MgO/Al_2_O_3_ samples at 32° angle (*θ*) of incidence of light (corresponds to *q* = 12.5 μm^−1^) for different thicknesses (*t* = 1, 5, 10 and 20 nm) of CoFeB layer.

The samples for this study are multilayer structures deposited on a Si (001) substrate with 700 nm SiO_2_ on it. The multilayer stacking is as follows: Ta (10)/Co_20_Fe_60_B_20_ (*t* = 1, 1.4, 2, 3, 5, 10 and 20)/MgO (2)/Al_2_O_3_ (10), where the numbers in parentheses denote the thickness of the layers in nanometers. The Co_20_Fe_60_B_20_ will be denoted as CoFeB in the rest of the manuscript. The multilayers were deposited by radio frequency (rf) sputtering at a base pressure of 10^−8^ Torr at room temperature. The details of the sample preparation can also be found in Refs.^45,46^. A schematic view of the sample is presented in Fig. 1a.

### Experimental setup

The thermally excited SAWs were studied using a six-pass, tandem Brillouin spectrometer (JRS Scientific Instruments), which ensures a contrast of ${10}^{15}$ refs.^47,48^. The incident light source was a Nd:YAG single-mode diode-pumped laser of 200 mW output power, which emits the second harmonic of wavelength *λ*_0_ = 532 nm (Excelsior, Spectra Physics). Measurements were performed in the backscattering geometry with *pp* polarization of light and the scattered light was collected using f/8 optics with a focal length of 58 mm. The solid angle of the lens was 0.63 steradians and the free spectral range was 20 GHz. A detailed description of the experimental setup can be found in Refs.^49,50^. BLS employs the inelastic scattering of incident photons from thermally excited phonons. The wavevector and frequency of the phonons in the studied samples can be characterized by measuring the projection of incident light wavevector *q* and frequency shift Δ*f* of scattered light. Due to momentum conservation in the scattering process, the wavevector of acoustic waves is equal to the projection of the incident light wavevector in the sample plane. Thus, the wavevector *q* of the acoustic waves can be expressed as:

1 $$q = \frac{4\pi \sin \theta }{{\lambda_{0} }}$$

where $\theta$ is the angle of incidence of light with the normal to the sample surface (Fig. 1a). The angle $\theta$ was varied in the range of 10°–80° to change *q* of the measured phonons in the range of 4–23 µm^−1^. The phase velocity (ν_*SAW*_) of SAWs can be correlated with $\theta$ by the following relation:

2 $$\Delta f_{SAW} = \frac{{2v_{SAW} \sin \theta }}{{\lambda_{0} }}$$

where $\Delta {f}_{SAW}$ is the Brillouin frequency shift for SAW^51^.

## Results

For transparent materials, the incident light is mostly scattered in the bulk of the material via the photoelastic coupling mechanism, while for opaque materials, the light is scattered from the surface of the material by the surface ripple mechanism. This fact provides an opportunity to probe SAWs in opaque materials. One of the criteria to identify the type of waves is that the Rayleigh-type SAWs in a given material must show a linear relationship between frequency and wavevector^49–52^. Such criteria perfectly work for homogeneous materials. However, in the case of multilayer films on the substrate, linear dispersion relations are not usually observed. These systems can be divided into two categories: slow-on-fast (when the velocity of the transverse bulk wave in the multilayer is smaller than that of the substrate) and fast-on-slow system (vice versa)^53^. The next criterion for Rayleigh SAW is that ν_*SAW*_ should be lower than that of the velocity (ν_*T*_) of the slowest transverse bulk waves.

The typical spectra for the studied sample are presented in Fig. 1b. For all the studied samples, the spectra show the Rayleigh waves (marked by RW in the figure). In some spectra, the low-intensity Sezawa waves were also observed (marked by arrows) and identified from FEM-based simulations, which will be discussed later.

**Figure 2:**
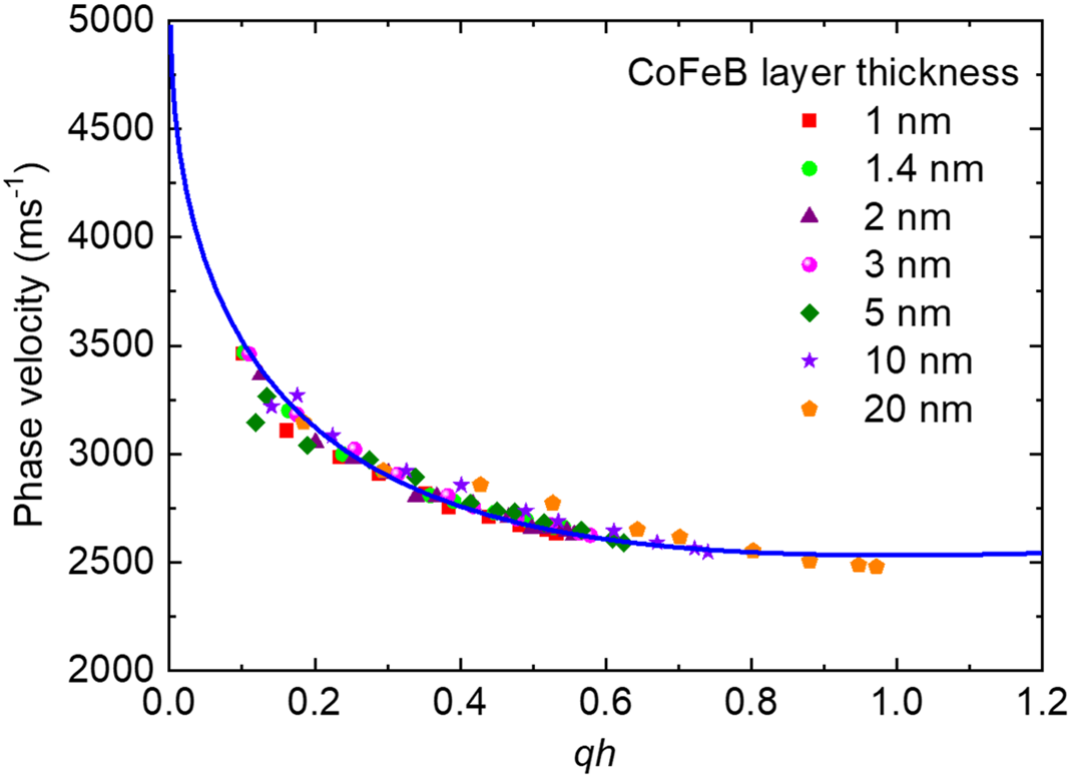
Phase velocity of Rayleigh SAWs obtained from the Si/SiO_2_/Ta/CoFeB(*t*)/MgO/Al_2_O_3_ samples for different wavenumbers. The solid curve represents the fit to an exponential decay function.

The measured phase velocity dispersion of Rayleigh SAWs as a function of wavevector × total layer thickness (i.e., *qh)* is shown in Fig. 2. Here, *q* is the wavevector (according to Eq. 1) and *h* is the total layer thickness of the multilayer system, i.e., $h=t_{\text{Ta}}+t_{\text{CoFeB}}+t_{\text{MgO}}+t_{\text{Al}_{2}\text{O}_{3}}$, where *t*_Ta_, *t*_CoFeB_, *t*_MgO_, $t_{\text{Al}_{2}\text{O}_{3}}$ are the thicknesses of Ta, CoFeB, MgO, Al_2_O_3_ layers, respectively.

The negative slope of phase velocity dispersion with *qh* suggests that the studied films can be classified as slow-on-fast systems. In these types of systems, the dispersion curves start with the transverse wave velocity (ν_T_) in the layer and asymptotically decrease to the Rayleigh wave velocity (ν_R_) in the layer deposited on the substrate^54^. By fitting the experimental data points with an exponential decay function, we extract that the ν_T_ (i.e., the velocity at *qh* = 0) in the studied layer is about 5000 m s^−1^, whereas the surface Rayleigh wave velocity in the overall stacking layer tends to be about 2500 m s^−1^. In our study, the penetration depth of Rayleigh waves is in the range of 250 nm to a few micrometers. According to the thickness of the SiO_2_ layer, we consider the substrate as Si/SiO_2_. To calculate the Rayleigh wave velocity, all the stacking layers should be considered as an effective layer instead of considering individual layers. This is worth mentioning here that the Rayleigh surface wave velocity in the overall stacking layer is quite different than in any individual layer of the stack. As the problem is not so trivial, we perform FEM-based simulations to determine the velocities of SAWs for large *qh* as described in section "Discussion".

## FEM simulation

**Figure 3:**
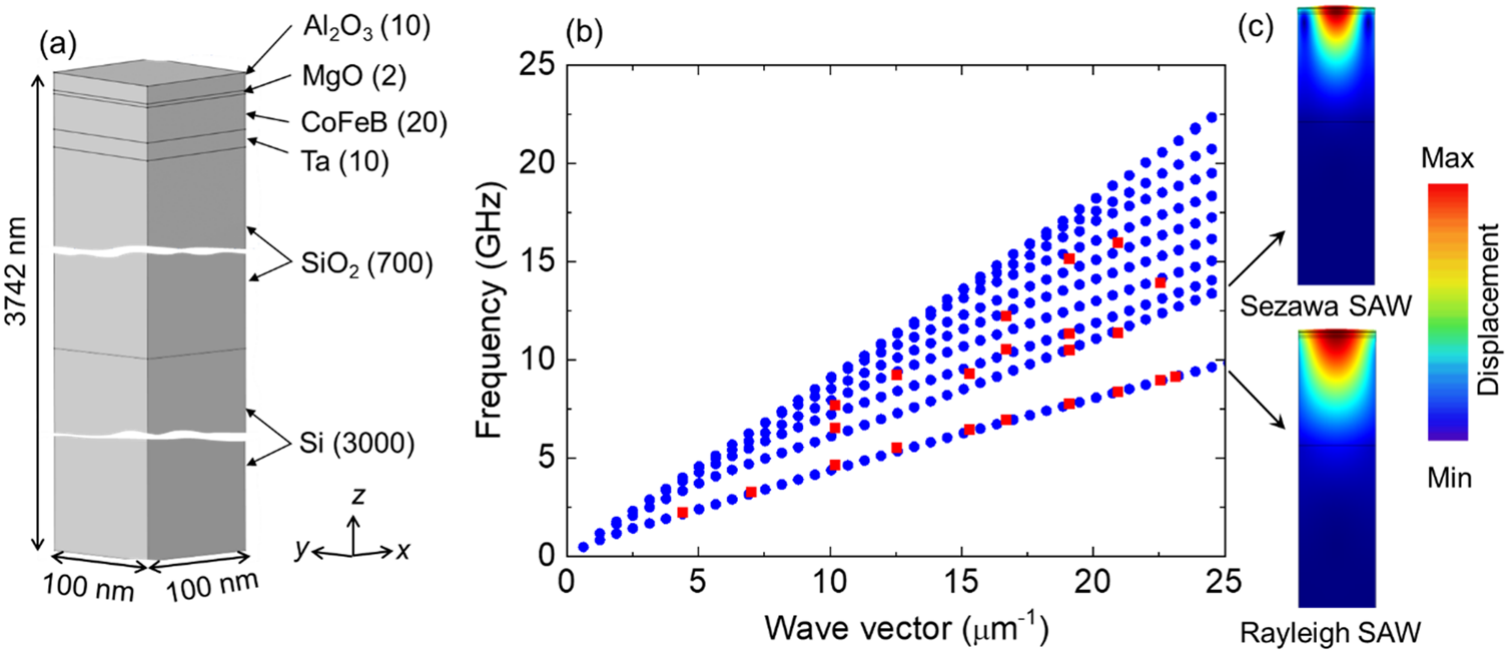
(**a**) The schematic diagram of the unit cell used for simulations. It consists of a long rectangular bar with dimensions 100 (x) × 100 (y) × 3742 (z) nm^3^ made of a multilayer. (**b**) Dispersion relation of SAWs obtained from the Si/SiO_2_/Ta/CoFeB (t = 20)/MgO/Al_2_O_3_ sample. Blue points represent the frequencies obtained from the FEM simulation, whereas red points are the frequencies obtained from the experiment. (**c**) The total displacement profile of SAW modes (Rayleigh wave, Sezawa wave) in studied samples is presented. The color scale is also provided.

To theoretically understand the dispersion character of the acoustic waves in the studied multilayers, FEM-based simulations were performed using COMSOL Multiphysics software^55^. The unit cell selected for simulations consists of a long rectangular bar with dimensions 100 (*x*) × 100 (*y*) × 3742 (*z*) nm^3^ made of a multilayer or effective layer (see Fig. 3a). We consider the substrate to be a uniform elastic half-space on which the multilayers with the determined thickness of each layer are present. The model assumes perfectly bonded and ideally flat parallel layers with zero interfacial thickness, uniform respective layer thickness, no interfacial roughness and defects, and uniform elastic properties within a given layer.

To reflect the conditions of the elastic semi-space, specific boundary conditions were applied. For the walls perpendicular to the free surface the Bloch-Floquet periodic boundary conditions were applied for each component of displacement:

3 $$\begin{aligned} & a\exp \left[ {i\left( {q_{x} x + q_{y} y} \right)} \right], \\ & b\exp \left[ {i\left( {q_{x} x + q_{y} y} \right)} \right], \\ \end{aligned}$$

where *a* and *b* denote the components of the displacement in the *x* and *y* direction in the cartesian coordinate system and *q*_*x*_ and *q*_*y*_ are the wavevector components given as:

4 $$\begin{aligned} q_{x} & = \frac{2\pi \cos \alpha }{{\lambda_{SAW} }}, \\ q_{y} & = \frac{2\pi \cos \beta }{{\lambda_{SAW} }}. \\ \end{aligned}$$

Here *α* and *β* are the angles made by the wavevector with *x* and *y* axes, respectively and λ_SAW_ is the wavelength of SAW. The exponential decay of the SAW amplitude was taken into account by applying a fixed boundary condition to the wall opposite to the free surface.

Figure 3b shows the dispersion relation of SAWs measured (red points) from the Si/SiO_2_/Ta/CoFeB (t = 20)/MgO/Al_2_O_3_ sample. In the studied multilayers both: the Rayleigh and Sezawa surface waves are visible. To calculate the dispersion characters of SAWs and understand the profile of the acoustic modes we first adapted the elastic parameters and densities of each layer as given in Table 1. Here, the parameters for all the materials, excluding CoFeB, are taken from indicated references. The elastic tensor components for CoFeB were calculated from the best fitting of frequency versus wavevector characters for all the multilayers with varying CoFeB thickness and it is described in the Discussion section. The calculated dispersion relation obtained from the simulations is shown in Fig. 3b by solid blue dots. A very good agreement is obtained between the experimental and simulation dispersion characters, especially for Rayleigh waves, using the estimated elastic parameters of CoFeB. We note here that the materials in the studied samples have different crystallographic symmetry, so the parameters have strickle characters to the materials and their symmetry. Two-dimensional maps of deformation for SAWs are presented in Fig. 3c. A difference in the deformation profile obtained for Rayleigh and Sezawa waves is clearly visible.

**Table 1:** Elastic constant and density of materials used for the simulation.

	Silicon^56^	Silicon dioxide^57^	Tantalum^58^	MgO^59^	Al_2_O_3_^60^
Elastic constants (in GPa)					
${c}_{11}$	166	75	260.91	295.9	466
${c}_{22}$	166	75	260.91	295.9	466
${c}_{33}$	166	75	260.91	295.9	506
${c}_{12}$	64	15	157.43	95.4	127
${c}_{13}$	64	15	157.43	95.4	117
${c}_{23}$	64	15	157.43	95.4	117
${c}_{44}$	80	30	81.82	153.9	235
${c}_{55}$	80	30	81.82	153.9	235
${c}_{66}$	80	30	51.74	153.9	169.5
${c}_{14}=-{c}_{24}={c}_{56}$	0	0	0	0	94
Density (in kg m^−3^)	2332	2650	16,600	3580	3950

**Figure 4:**
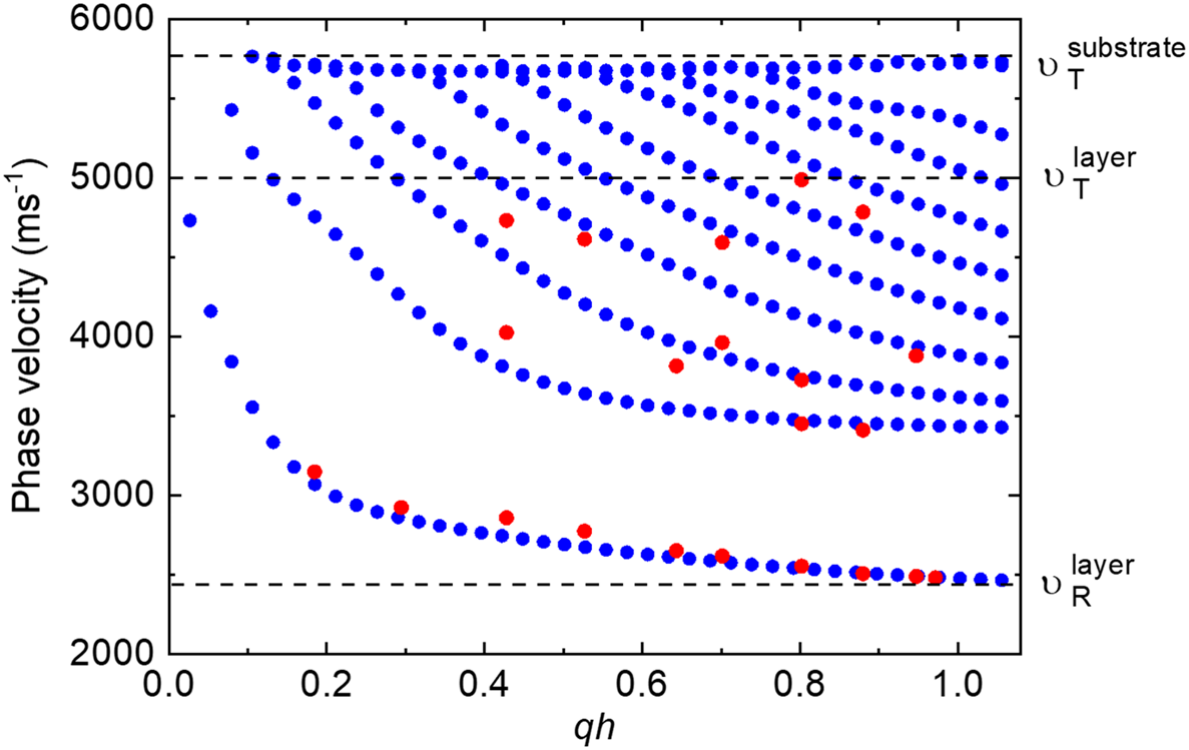
Phase velocity dispersion of SAWs, obtained from Si/SiO_2_/Ta/CoFeB (t = 20)/MgO/Al_2_O_3_ sample, are plotted as a function of qh. Red dots are experimental results obtained from BLS measurements, whereas blue dots are simulation results performed by FEM.

In Fig. 4, the phase velocity dispersions of SAWs for Si/SiO_2_/Ta/CoFeB (t = 20)/MgO/Al_2_O_3_ film are plotted. Here, the red points represent the experimental results, whereas the blue points represent the results obtained from FEM simulations. The dispersion plots reconfirm that the studied multilayer is a slow-on-fast system. The main criterion of the above classification is ${v}_{T}^{layer}<{\sqrt{2}\cdot v}_{T}^{substrate}$ for slow-on-fast systems, while the criterion for the fast-on-slow system is ${v}_{T}^{layer}>{\sqrt{2}\cdot v}_{T}^{substrate}$ according to Farnell and Adler^53^. For slow-on-fast systems, the presence of an elastically soft layer on an elastically hard substrate leads to the reduction of Rayleigh SAWs velocity and formation of higher-order modes known as Sezawa waves, whose displacement profile is presented in Fig. 3c. The simulated phase velocity dispersion graphs allow us to estimate the phase velocities of transverse bulk waves in the layer and in the substrate, which are about 5000 m s^−1^ and 5740 m s^−1^, respectively. The phase velocity of Rayleigh SAW in the multilayer is estimated to be 2350 m s^−1^. Here, the substrate is defined as Si/SiO_2,_ and the layer is defined as Ta/CoFeB/MgO/Al_2_O_3_. The estimated values of velocities for all studied samples are the basis for calculations of the elastic parameters of the CoFeB layer and effective layers.

## Discussion

For an in-depth understanding of the effect of CoFeB layer thickness on the velocity of SAWs in the studied materials, some additional simulations on SAW propagation were performed by FEM. The penetration depths of Rayleigh-type SAWs in the materials partly depend on the wavevector. We have previously mentioned that the substrate must be taken into account while calculating the characters of SAWs, especially for smaller wavevectors. As the penetration depth is way larger than the total thickness of the multilayer (which is a maximum of 42 nm for a multilayer with 20 nm thick CoFeB layer), the studied multilayers Ta/CoFeB/MgO/Al_2_O_3_ can be treated as an effective layer on the substrate. In that way, the effective elastic parameter of the multilayer can be estimated. To do so we first calculate the elastic parameters of CoFeB under two conditions. The best agreement between the experimental results and FEM simulations is treated as the first condition. The second condition is to fulfill the Cauchy relations^61,62^. We started performing simulations with the value of elastic tensor components of CoFeB as proposed in Ref.^23^. However, these parameters didn’t fulfill our first condition. So, we modified the parameters in such a way that it fulfills both the above-mentioned conditions. The estimated elastic tensor components for CoFeB are (in GPa):

$$\left( {\begin{array}{*{20}c} {210} & {130} & {130} & 0 & 0 & 0 \\ {130} & {210} & {130} & 0 & 0 & 0 \\ {130} & {130} & {210} & 0 & 0 & 0 \\ 0 & 0 & 0 & {45} & 0 & 0 \\ 0 & 0 & 0 & 0 & {45} & 0 \\ 0 & 0 & 0 & 0 & 0 & {45} \\ \end{array} } \right)$$

**Figure 5:**
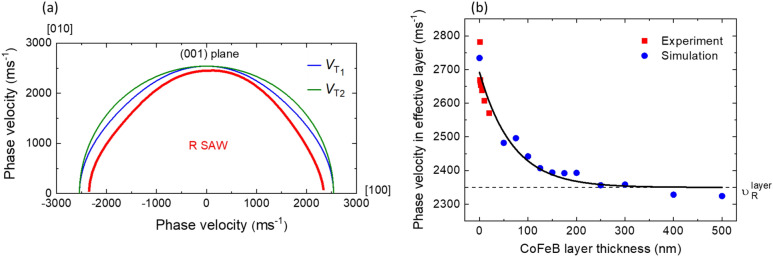
The phase velocity of bulk acoustic waves (T_1_, T_2_—transverse waves) and Rayleigh wave (R SAW) for CoFeB (**a**); and SAWs (**b**) obtained for effective layers with different thicknesses of CoFeB.

Based on these parameters, the calculated phase velocities of bulk acoustic modes and Rayleigh SAW, which propagate on the surface (001) are presented in Fig. 5.

The value of Rayleigh SAW in CoFeB, calculated according to FEM simulations, is around 2350 m s^−1^ (Fig. 5a). Interestingly, the same value is also obtained by plotting phase velocity in the effective layer as a function of CoFeB thickness and fitting with exponential function as depicted in Fig. 5b. These results suggest that the layers in the studied samples can be treated as an effective layer. The CoFeB layer has cubic symmetry, but when the CoFeB is included in a multilayer system it becomes more convenient to use Young’s modulus which can be calculated from the estimated elastic tensors of CoFeB. In our case, Young’s modulus $E$, as described in^63^, is given as:

5 $$E = \frac{{\sigma_{33} }}{{\varepsilon_{33} }} = \frac{1}{{s_{33}^{^{\prime}} }}$$

where ${s}_{33}^{^{\prime}}$ is the component of stiffness in the measurement plane of the system (in Voigt notation). For a material with cubic symmetry, the ${s}_{33}^{^{\prime}}$ component of stiffness is given as^63^:

6 $${s}_{33}^{^{\prime}}={s}_{3333}^{^{\prime}}={s}_{11}-({s}_{11}-{s}_{12}-\frac{1}{2}{s}_{44})[1-{\mathrm{cos}}^{4}\varphi {\mathrm{sin}}^{4}\psi -{\mathrm{sin}}^{4}\varphi {\mathrm{sin}}^{4}\psi -{\mathrm{cos}}^{4}\psi ]$$

where ${s}_{11}, {s}_{12},$ and ${s}_{44}$ are the components of stiffness in the crystal plane of the system and $\varphi$ and $\psi$ are the two arbitrary rotation angles to transform the cubic crystal coordinate system into a lattice plane system. The relations among stiffness components ${s}_{11}, {s}_{12},$ and ${s}_{44}$ and elastic constants ${c}_{11}, {c}_{12}$ and ${c}_{44}$ are given as:

7 $$\begin{aligned} s_{11} & = \frac{{c_{11} + c_{12} }}{{\left( {c_{11} - c_{12} } \right)\left( {c_{11} + 2c_{12} } \right)}} \\ s_{12} & = \frac{{ - c_{12} }}{{\left( {c_{11} - c_{12} } \right)\left( {c_{11} + 2c_{12} } \right)}} \\ s_{44} & = 1/c_{44} . \\ \end{aligned}$$

Using Eqs. (5)–(7), Young’s modulus $E$ can be written as:

8 $$E = \frac{{2\left( {c_{11} - c_{12} } \right)\left( {c_{11} - c_{12} } \right)c_{44} }}{{2\left( {c_{11} + c_{12} } \right) - \left( {c_{11} + 2c_{12} } \right)\left( {2c_{44} - c_{11} + c_{12} } \right)\left[ {1 - \cos^{4} \varphi \sin^{4} \psi - \sin^{4} \varphi \sin^{4} \psi - \cos^{4} \psi } \right]}}$$

**Figure 6:**
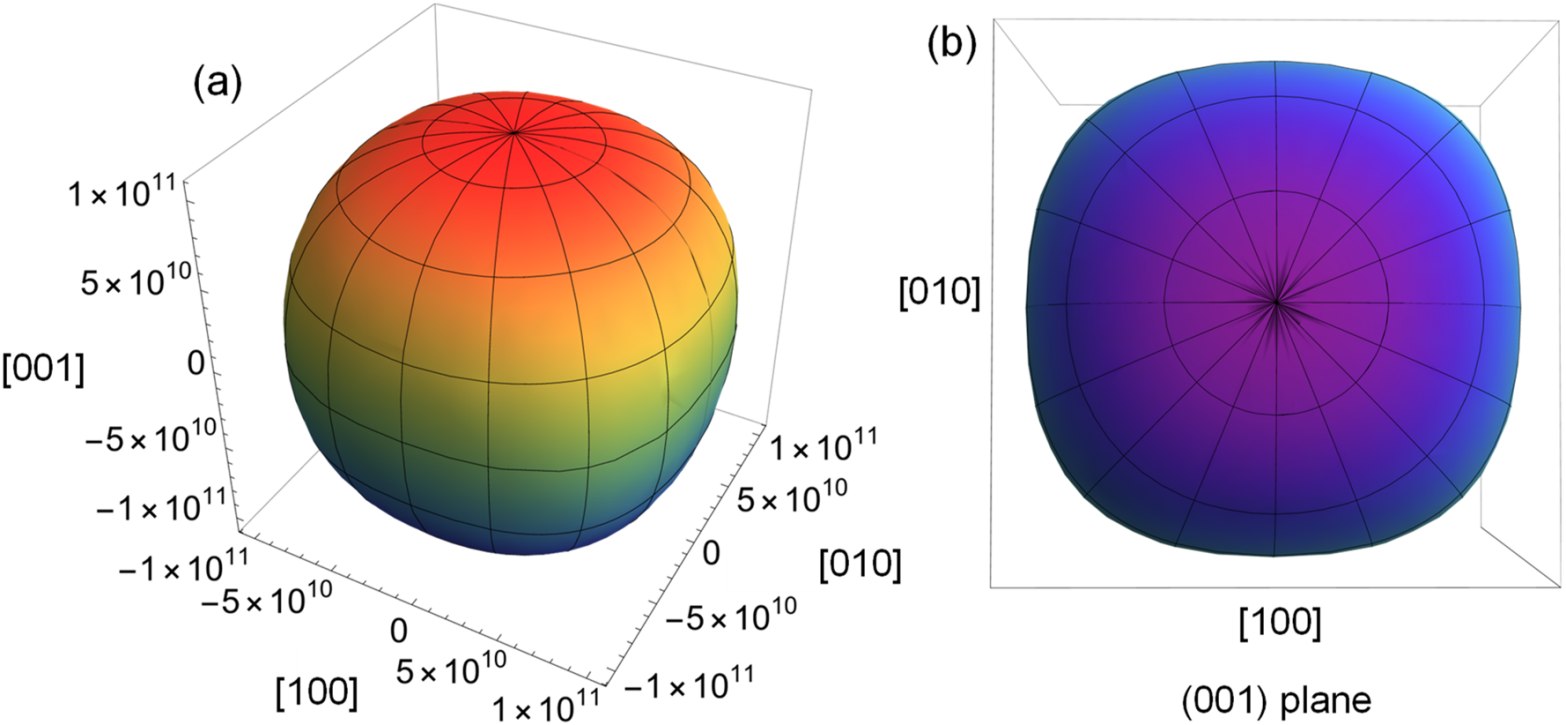
(**a**) The 3D view and (**b**) view in (001) plane of calculated Young’s modulus $(E$) of CoFeB.

With the estimated elastic components, Young’s modulus $E$ is plotted for CoFeB as shown in Fig. 6.

We calculate the elastic parameters of the effective layers for varying thicknesses of the CoFeB layer. This is done by adapting the proportion method, where elastic parameters of individual layers are multiplied by the volume fraction of the effective layer, added thereafter, and averaged out with the total volume^64,65^. There is no unique way to do this, and different literatures propose different procedures^66,67^. We used ELATE^68^, which is an open-source online tool for the analysis of elastic tensors, to calculate Young’s modulus and Poisson’s ratio of each layer. Then, we calculated their weighted average based on the thickness of each layer.

**Figure 7:**
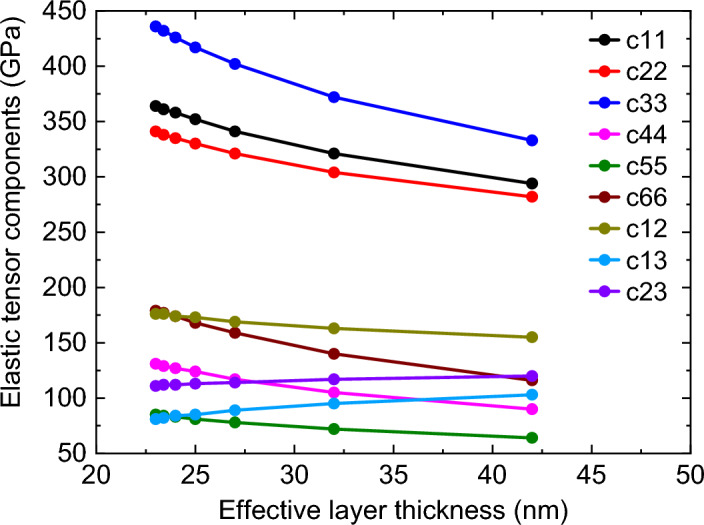
Calculated values of elastic tensor components for effective layer as a function of total or effective layer thickness *h*.

**Figure 8:**
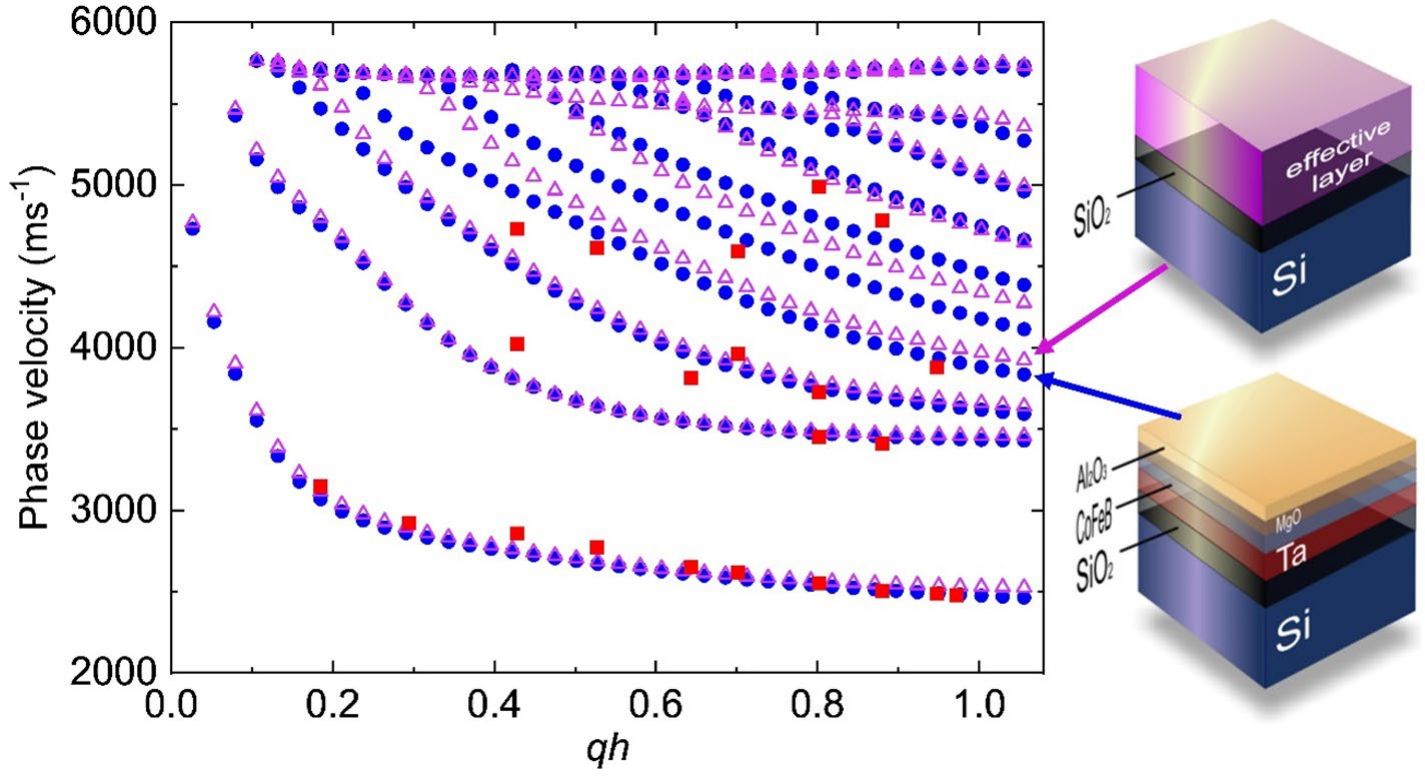
The phase velocity of SAWs obtained from the Si/SiO_2_/Ta/Co_20_Fe_60_B_20_ (*t* = 20)/MgO/Al_2_O_3_ samples as individual layers (circles) and as effective layer (triangles). The square points represent the phase velocity obtained from the experiment.

Figure 7 represents the plot of the calculated values of elastic tensors of the effective layer as a function of total layer thickness (*h*), which shows a significant variation of effective elastic parameters with *h*. The accuracy of the elastic tensor values is in the range of 5 GPa. Next, we compare the phase velocity dispersion of SAWs calculated by considering the elastic parameters of individual layers and the elastic parameters of the effective layer (Fig. 8). Both results show great agreement with each other.

Finally, we calculate Young’s modulus and Poisson’s ratio of the effective layer. A multilayer system can consist of various layers with different crystallographic symmetries and different numbers of independent elastic tensor components. However, for the sake of simplicity, we can treat the effective layer as isotropic and define elastic parameters and densities for it. Although, different studies propose different procedures^69,70^, we employ above mentioned weighted average technique and the calculated values of Young’s modulus and Poisson’s ratio are presented in Table 2. These values will be very useful to analyze the coupling between phonons and magnons.

**Table 2:** Young’s modulus and Poisson’s ratio for uniform effective layers (isotropic).

CFB layer thickness (nm)	Density (kg m^−3^)	Young’s modulus (GPa)	Poisson’s ratio
1	9549	249	0.31
1.4	9505	249	0.31
2	9442	243	0.31
3	9343	238	0.31
5	9167	228	0.32
10	8824	210	0.33
20	8382	187	0.34

## Conclusions

In conclusion, we have studied frequency versus wavevector dispersion of thermally excited SAWs in Si/SiO_2_/Ta/CoFeB(*t*)/MgO/Al_2_O_3_ heterostructures with varying CoFeB thickness (*t*) from 1 to 20 nm. The thermally excited acoustic phonons are detected by employing Brillouin light spectroscopy in backscattering geometry. Both Rayleigh-type and Sezawa-type SAWs are detected in the experiment and their frequency versus wavevector dispersions are measured up to a wavevector of 23 μm^−1^. The experimental results are corroborated by finite element method based COMSOL simulations. The simulated mode profiles of Rayleigh and Sezawa waves show very distinct spatial features. We calculate the elastic tensor parameters for the CoFeB layer from the best agreement of simulation results with the experiment data points. We further estimate the effective elastic parameters (elastic tensors, Young’s modulus, Poisson’s ratio) of the whole stacks for varying CoFeB thickness by considering the whole stack as a uniform layer. It is observed that the effective elastic parameters significantly vary with the CoFeB layer thickness. Interestingly, the simulation results, either considering elastic parameters of individual layers or considering effective elastic parameters of whole stacks, show good agreement with the experimental results. These extracted elastic parameters will be very useful to understand the interaction of phonons with other quasiparticles such as magnons and skyrmions in a similar type of heterostructures.

## Data Availability

The dataset used and/or analyzed during the current study will be made available by the corresponding author on reasonable request.
